# Supplementary material for: Decoupled recovery of ecological communities after reclamation
Source: PeerJ. 2019 Jun 21;7:e7038. doi: 10.7717/peerj.7038 (PMC6590388; doi:10.7717/peerj.7038)
Supplement: Table S1 — Designations include whether a species is native (N) or exotic (E), with invasive (I) or ruderal (R) status indicated in parentheses where appropriate. Functional groups include cactus (C), forb (F), grass (G), legume (L), sedge (Se), shrub (S) and sub-shrub (Sub). Asterisks denote an indicator species determined from indicator species analysis. [file peerj-07-7038-s001.docx]

| Name | Authority | Family | Designation | Functional Group |
| --- | --- | --- | --- | --- |
| *Achillea millefolium* | L. | Asteraceae | N | F |
| *Anemone canadensis* | L. | Ranunculaceae | N | F |
| *Antennaria sp.* |  | Asteraceae | N | F |
| *Artemisia cana* | Pursh | Asteraceae | N | S |
| *Artemisia frigida* | Willd. | Asteraceae | N | Sub |
| *Aristida purpurea* | Nutt. | Poaceae | N | G |
| *Symphyotrichum oblongifolium* | (Nutt.) G.L. Nesom | Asteraceae | N | F |
| *Bouteloua curtipendula* | (Michx.) Torr. | Poaceae | N | G |
| *Bouteloua gracilis** | (Willd. Ex Kunth) Lag. Ex Griffiths | Poaceae | N | G |
| *Carex duriuscula* | C.A. Mey. | Cyperaceae | N | Se |
| *Carex filifolia** | Nutt. | Cyperaceae | N | Se |
| *Calamovilfa longifolia* | (Hook.) Scribn. | Poaceae | N | G |
| *Dalea purpurea* | Vent. | Fabaceae | N | L |
| *Distichlis spicata** | (L.) Greene | Poaceae | N | G |
| *Echinacea angustifolia* | DC. | Asteraceae | N | F |
| *Elymus trachycaulus** | (Link) Gould ex Shinners | Poaceae | N | G |
| *Geum triflorum* | Pursh | Rosaceae | N | F |
| *Grindelia squarrosa* | (Pursh) Dunal | Asteraceae | N (R) | F |
| *Gutierrezia sarothrae* | (Pursh) Britt. & Rusby | Asteraceae | N | Sub |
| *Hesperostipa comata* | (Trin. & Rupr.) Barkworth | Poaceae | N | G |
| *Helianthus paucifloris* | Nutt. | Asteraceae | N | F |
| *Juniperus horizontalis* | Moench | Cupressaceae | N | S |
| *Koeleria macrantha* | (Ledeb.) Schult. | Poaceae | N | G |
| *Koeleria sp.* |  | Poaceae | N | G |
| *Linum sp.* |  | Linaceae | N | F |
| *Lygodesmia juncea* | (Pursh) D. Don ex Hook. | Asteraceae | N | F |
| *Muhlenbergia cuspidata* | (Torr. ex Hook.) Rydb. | Poaceae | N | G |
| *Nassella viridula* | (Trin.) Barkworth | Poaceae | N | G |
| *Opuntia fragilis* | (Nutt.) Haw. | Cactaceae | N | C |
| *Opuntia polyacantha* | Haw. | Cactaceae | N | C |
| *Pascopyrum smithii* | (Rydb.) A. Love | Poaceae | N | G |
| *Pediomelum argophyllum* | (Pursh) J. Grimes | Fabaceae | N | L |
| *Phlox hoodii* | Richardson | Polemoniaceae | N | F |
| *Plantago patagonica* | Jacq. | Plantaginaceae | N | F |
| *Polygala alba* | Nutt. | Polygalaceae | N | F |
| *Ratibida columnifera* | (Nutt.) Wooton & Standl. | Asteraceae | N | F |
| *Rosa arkansana* | Porter | Rosaceae | N | Sub |
| *Schizachyrium scoparium* | (Michx.) Nash | Poaceae | N | G |
| *Solidago rigida* | L. | Asteraceae | N | F |
| *Sphaeralcea coccinea* | (Nutt.) Rydb. | Malvaceae | N | F |
| *Sporobolus cryptandrus* | (Torr.) A. Gray | Poaceae | N | G |
| *Symphoricarpos sp.* |  | Caprifoliaceae | N | S |
| *Agropyron cristatum** | (L.) Gaertn. | Poaceae | E (I) | G |
| *Bromus inermis* | Leyss. | Poaceae | E (I) | G |
| *Bromus japonicus* | Thunb. | Poaceae | E (I) | G |
| *Bassia scoparia* | (L.) A.J. Scott | Chenopodiacea | E (I) | F |
| *Medicago lupulina** | L. | Fabaceae | E (R) | L |
| *Melilotus officinalis* | (L.) Pall. | Fabaceae | E | L |
| *Poa compressa* | L. | Poaceae | E (I) | G |
| *Poa pratensis* | L. | Poaceae | E (I) | G |
| *Taraxacum officinale* | (L.) Weber ex F.H. Wigg | Asteraceae | E (R) | F |
| *Tragopogon dubius* | Scop. | Asteraceae | E (R) | F |
