# Supplementary material for: Decoupled recovery of ecological communities after reclamation
Source: PeerJ. 2019 Jun 21;7:e7038. doi: 10.7717/peerj.7038 (PMC6590388; doi:10.7717/peerj.7038)
Supplement: Table S2 — Exotic species in bold and functional groups denoted by F (forb), G (grass) or S (shrub). Ruderal plant species are denoted by ‡ and invasive species are denoted by *. [file peerj-07-7038-s002.docx]

| Transect type | Species | Functional group | Indicator Value | P-Value |
| --- | --- | --- | --- | --- |
| 150 m | *Carex filifolia* (Nutt.) | G | 0.45 | 0.026 |
| 50 m | *Bouteloua gracilis* ([Willd. ex Kunth] Lag. ex Griffiths) | G | 0.57 | 0.004 |
| Reclaim | *Elymus trachycaulus* ([Link] Gould ex Shinners) | G | 0.62 | 0.001 |
|  | ***Agropyron cristatum**([L.] Gaertn.)** | G | 0.6 | 0.002 |
|  | ***Medicago lupulina‡* (L.)** | F | 0.59 | 0.001 |
|  | *Distichlis spicata* ([L.] Greene) | G | 0.53 | 0.005 |
