# Supplementary material for: Decoupled recovery of ecological communities after reclamation
Source: PeerJ. 2019 Jun 21;7:e7038. doi: 10.7717/peerj.7038 (PMC6590388; doi:10.7717/peerj.7038)
Supplement: Table S3 — Bold text denotes significant differences between sampling location (letters denote post-hoc test groupings; see text). [file peerj-07-7038-s003.docx]

| Nematode trophic group | 150 m | 50 m | Reclaim |
| --- | --- | --- | --- |
| Bacterivores | 5531 ± 582 | 2389 ± 638 | 2212 ± 591 |
| Fungivores | 2337 ± 234 | 2474 ± 373 | 1868 ± 175 |
| Root herbivores | 7056 ± 971 | 6203 ± 720 | 4903 ± 766 |
| **Omnicarnivores** | **3084 ± 316 a** | **2586 ± 241 ab** | **2162 ± 266 b** |
| **Total** | **18007 ± 1614 a** | **17305 ± 370 ab** | **14153 ± 1166 b** |
