# Supplementary material for: Decoupled recovery of ecological communities after reclamation
Source: PeerJ. 2019 Jun 21;7:e7038. doi: 10.7717/peerj.7038 (PMC6590388; doi:10.7717/peerj.7038)
Supplement: Table S4 [file peerj-07-7038-s004.docx]

| **Response variable** | **Predictor variable** | **Estimate ± SE** | **Z-statistic** | **P** |
| --- | --- | --- | --- | --- |
| Nematode community | Bare ground | -0.026 ± 0.01 | -2.684 | 0.007 |
| Ruderal plants | Salt concentrations | 1.802 ± 0.889 | 2.027 | 0.043 |
|  | Bare ground | 0.112 ± 0.076 | 1.467 | 0.142 |
| Native perennials | Exotic perennials | -1.316 ± 0.191 | -6.892 | < 0.0001 |
|  | SOM | 0.057 ± 0.035 | 1.622 | 0.105 |
|  | Bare ground | -0.003 ± 0.004 | -0.759 | 0.448 |
| Exotic perennials | SOM | 0.053 ± 0.036 | 1.488 | 0.137 |
|  | Bare ground | 0.004 ± 0.002 | 1.806 | 0.071 |
