# Supplementary figures and images for: Decoupled recovery of ecological communities after reclamation
Source: PeerJ. 2019 Jun 21;7:e7038. doi: 10.7717/peerj.7038 (PMC6590388; doi:10.7717/peerj.7038)

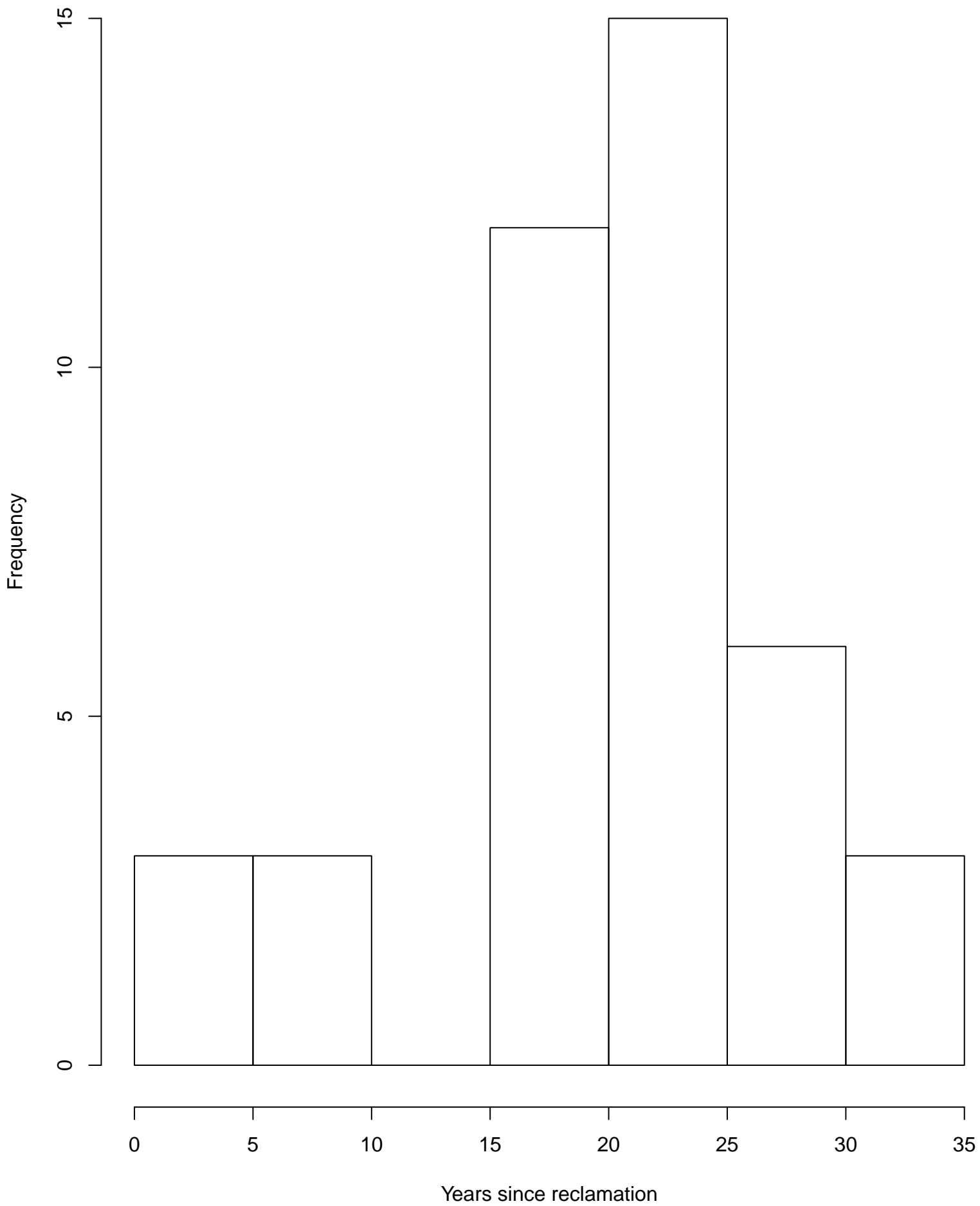

Supplement: Figure S1 [file peerj-07-7038-s005.pdf]

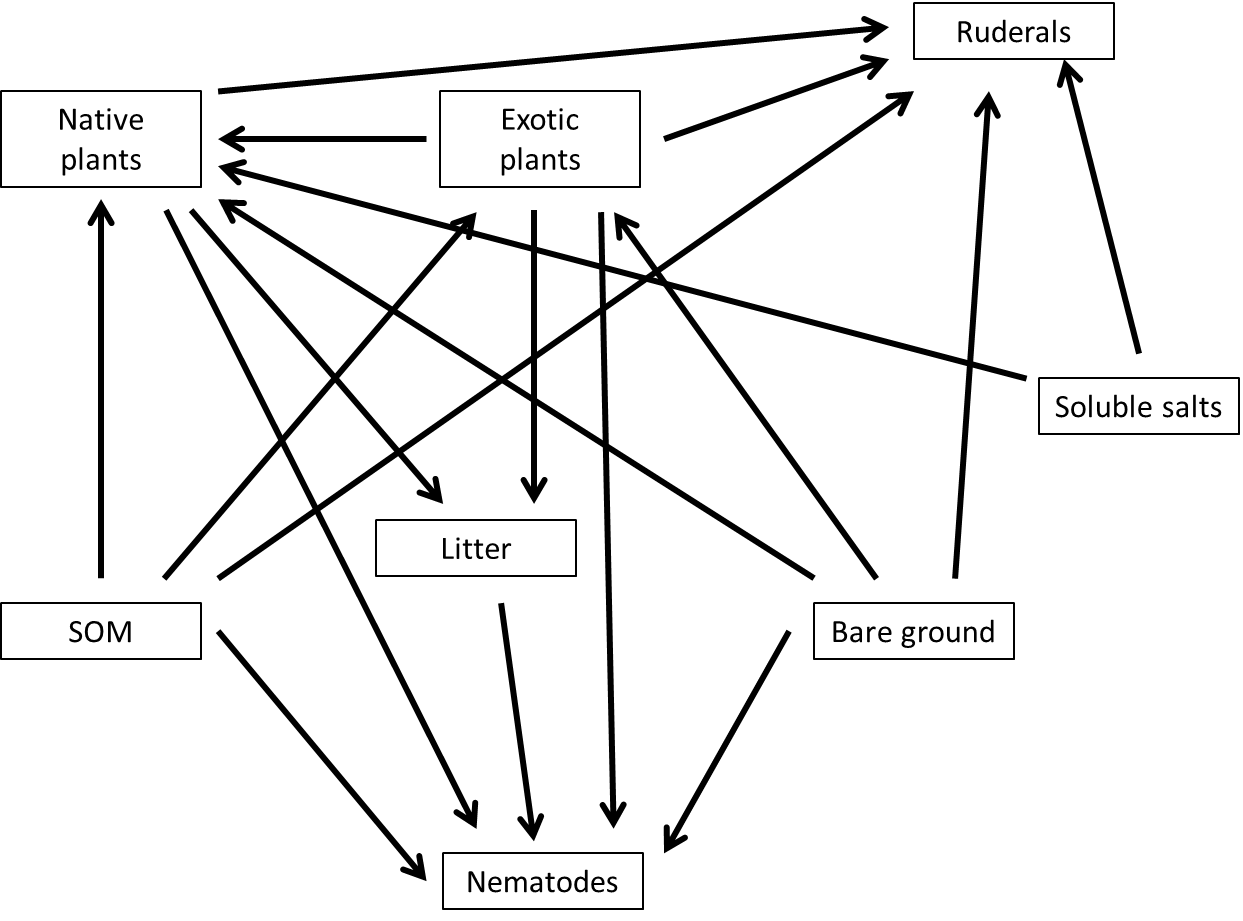

Supplement: Figure S2 — The conceptual model is constructed on hypotheses related to environmental filtering (impacts of soluble salts) and resource availability (SOM, litter and plant factors for nematodes). [file peerj-07-7038-s006.png]
